# Luminescent ruthenium(II) polypyridyl complexes acted as radiosensitizer for pancreatic cancer by enhancing radiation-induced DNA damage

**DOI:** 10.7150/thno.34015

**Published:** 2019-09-18

**Authors:** Yuyang Zhou, Ying Xu, Lunjie Lu, Jingyang Ni, Jihua Nie, Jianping Cao, Yang Jiao, Qi Zhang

**Affiliations:** 1 School of Chemistry, Biology and Materials Engineering, Jiangsu Key Laboratory of Environmental Functional Materials, Suzhou University of Science and Technology, Suzhou, Jiangsu, 215009, P. R. China.; 2 School of Radiation Medicine and Protection, State Key Laboratory of Radiation Medicine and Protection, Medical College of Soochow University, Suzhou, Jiangsu 215123, P. R. China.; 3 Department of Radiation Physics, Qingdao Central Hospital, Qingdao, Shandong, 266000, P. R. China.

**Keywords:** radiosensitizing, ruthenium(II), 4,7-diphenyl-1,10-phenoline, polypyridyl, pancreatic cancer.

## Abstract

**Background**: Pancreatic cancer is a highly lethal malignancy which ranks 4th most common cause of cancer death in US and 6th in China. Novel drugs are required to improve the survival and prognosis of patients.

**Methods**: Ruthenium(II) complexes with variation number of DIP ligand were synthesized and further adopted as radiosensitizer for pancreatic cancer. The influence of ruthenium(II) complexes on cell behaviors and tumor growth were investigated. The DNA binding affinity of ruthenium(II) complexes and plasmid was measured by using agarose gel electrophoresis.

**Results**: Luminescent ruthenium(II) complex can rapidly enter into cell nuclei and consequently combine with DNA, resulting in the enhanced DNA damage induced by X-ray irradiation. Upon intratumoral injection of ruthenium(II) complex, excellent tumor growth inhibition was accomplished under ionizing radiation of human pancreatic cancer xenograft nude mice.

**Conclusions**: Taken together, our study suggest that the ruthenium(II) polypyridyl complexes can effectively enhance radiation-induced DNA damage, which is likely to benefit the imaging-guided cancer radio-chemotherapy.

## Introduction

Pancreatic cancer is a highly lethal malignancy which accounts for more than 200,000 deaths every year world widely 1. In China, pancreatic cancer ranks 6th in the mortality rate of human malignant tumors 1, 2. Owing to its highly metastatic potential, no more than 20% of patients are suitable for surgical resection at first diagnosis, and the 5-year survival rate of this disease is about 4% due to poor prognosis of conventional adjuvant radiotherapy and chemotherapy 1.

**Figure 1:**
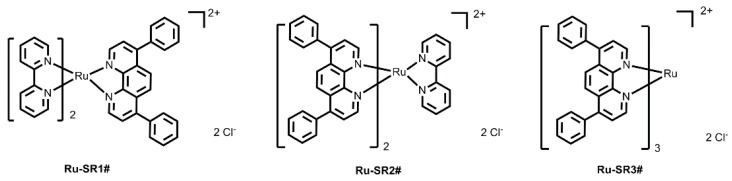
The chemical structures of luminescent ruthenium(II) complexes with different number of DIP ligand.

In order to improve the prognosis of pancreatic cancer, new drugs and therapeutics are urgently desired. Taking advantage of the enhanced photoelectric and Compton effects of metal atoms 3, metal-based complex is one kind of the important radiosensitizer agents for radio-chemotherapy, such as cisplatin, carboplatin and other platinum-based drugs. However, platinum-based drugs have some serious side effects and poor tumor selectivities in clinical treatment of cancer patients. Therefore, exploring other metal-based drugs as radiosensitizer is extremely imperative. In the past few decades, ruthenium complexes have some favorable properties over other metal-based complexes and have been identified as one of the most potential alternatives to the commercial platinum-based radiosensitizers. Chan et. al. initially reported the radiosensitizing abilities of RuCl_2_(DMSO)_2_(4-nitroimidazole) through *in vitro* experiments in 1986 4. In 2016, Carter et. al. reported the radiosensitizing abilities of Ru(II) arene compound (abbreviated as [(η^6^-arene)Ru^II^(en)Cl]^+^) on human colorectal cancer cells 5. Meanwhile, Chen group reported the radiosensitization of ruthenium complexes comprising benzimidazolyl group 6, 7 and selenium element 8. Very recently, Gill and co-workers investigated the radiosensitizing properties of substitutionally inert ruthenium polypyridyl complex comprising dipyridophenazine (dppz) 9, 10. By tuning the ligands 11, 12, ruthenium complexes can emit long-lived phosphorescence with high quantum efficiency and large Stokes' shifts, in favor of monitoring of the drug luminescent signals during the cancer therapeutic process 13-16. Notably, the long lifetime of ruthenium complex makes it possible to detect its phosphorescence by using time-resolved spectroscopy 17, distinct from the relatively fast auto-fluorescence generated by the organism. Very recently, TLD-1433, one of the typical polypyridyl-based ruthenium(II) complex, entered phase IB clinical trials as a photodynamic therapy (PDT) agent in patients with bladder cancer 18. 4,7-diphenyl-1,10-phenoline (DIP) has comparatively bulk aromatic groups and is much more lipophilic compared with other polypyridyl ligands, which may contribute to design ruthenium(II)-polypyridyl complexes as anticancer agents 19-22. However, there are almost few detailed reports to investigate the potential possibilities of DIP ligand in terms of radiosensitizing human cancer for radio-chemotherapy. Herein, three coordinatively saturated and substitution inert ruthenium(II) complex with variation number of DIP ligand are deliberately to design and synthesized (shown in Figure 1). The intake process of ruthenium(II) complex of pancreatic cancer cells and the interaction between ruthenium(II) complex and DNA were investigated under X-ray exposure. Furthermore, the BALB/C nude mice with intratumoral cell tumor was constructed to evaluate the radiosensitization of ruthenium(II) complexes with DIP ligand. Our findings open an avenue for the development of ruthenium(II) polypyridyl complex-based radiosensitizer for cancer therapy.

## Results and Discussion

### Design and Synthesis

Previous studies have verified that various kinds of luminescent metal complex with DIP ligand have excellent DNA binding abilities 20, 23-26. Taking into account the advantages of ruthenium (II) complexes in anticancer and the excellent photophysical properties of substitutionally inert ruthenium(II) complexes with polypyridyl ligand, three luminescent ruthenium(II) complexes with different number of DIP ligands (shown in Figure 1) are rationally designed and successfully synthesized in this work.

The synthetic procedures for these complexes are all detailed described in Experimental section. Briefly, the synthesis of Ru-SR1# used the commercial available starting material of *cis*-bis-(2,2'-bipyridine)dichlororuthenium(II) dihydrate (abbreviated as *cis*-Ru(bpy)_2_Cl_2_) and only need one step of the reaction between *cis*-bis-(2,2'-bipyridine)dichlororuthenium(II) dihydrate and DIP ligand with molar ratio of 1:1 in methanol solution. While the synthesis of Ru-SR2# and Ru-SR3# is a little complicated. In details, the intermediate product of *cis*-bis-(4,7-diphenyl-1,10-phenanthroline)dichlororuthenium(II) dihydrate (abbreviated as *cis*-Ru(DIP)_2_Cl_2_) was firstly synthesized through the reaction between ruthenium(III) chloride hydrate and DIP ligand with molar ratio of 1:2 in DMF solution. The as-prepared crude material was purified just through washing with *n*-hexane, cold acetone and cold methanol in sequence. Without further purification, the intermediate was used further to react with 4,4'-bipyridine (bpy) or DIP (molar ratio of 1:1) and the luminescent complexes Ru-SR2# and Ru-SR3# were successfully synthesized, respectively. According to Figure S10 and Table S1-S3, these ruthenium(II) complexes displayed red emission (~615 nm) with a lifetime of around 6 µs. As expected, along with the increasing of DIP ligand number, the lipophilic of corresponding complexes was augmented (see Table S1). The experimental photophysical properties was in according with the theoretical calculation of ground and excited states by DFT and TD-DFT method.

### Ruthenium complexes exhibit selective cytotoxicity on human pancreatic cancer cells

**Figure 2:**
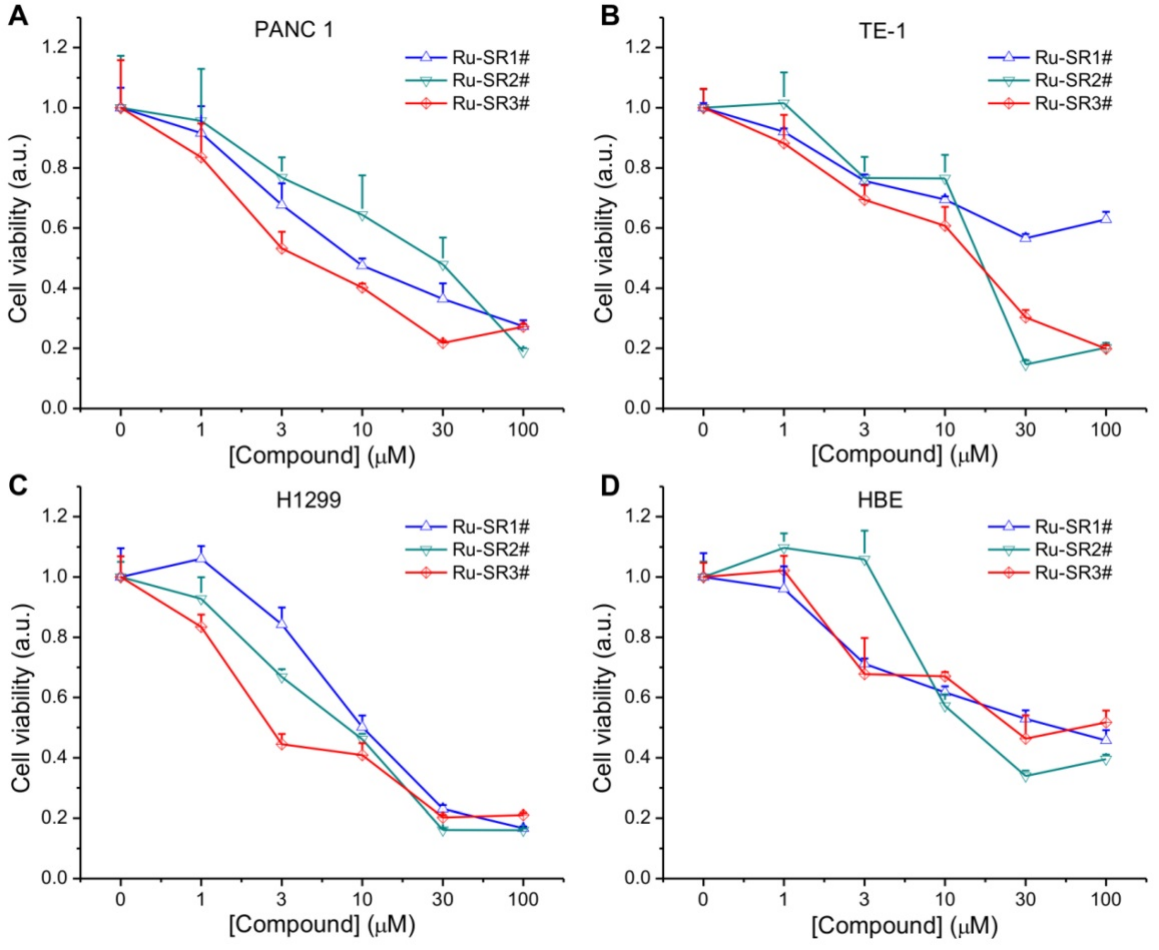
** Cell viability of ruthenium(II) complexes.** The PANC 1(**A**), TE-1 (**B**), H1299 (**C**) and HBE (**D**) cell lines were incubated with different concentration of ruthenium(II) complexes for 24 h, and the cell viabilities were measured in an cell counting kit-8 (CCK8) assay.

To evaluate the anti-pancreatic cancer potential of ruthenium complexes and possible universal anti-cancer therapeutics, human pancreatic cancer cell line PANC 1, esophageal cancer cell line TE-1, non-small lung cancer cell line H1299 and normal human bronchi epithelial cell line HBE, were exposed to different dose of ruthenium complexes (1, 3, 10, 30, 100 μmol/L) for 24 h, then proceeded to CCK8 viability assay. As shown in Fig 2A, 2B, 2C and Table 1, among these ruthenium complex sensitizers, Ru-SR3# illustrated better anti-proliferative effects on PANC 1 cells, represented as the much lower IC50 (<10 μM), comparing to Ru-SR1# and Ru-SR2#. For TE-1 and H1299 cells, Ru-SR3# also exhibited a high anticancer activity, indicating a broad-spectrum anticancer effect 10. Taken together the data shown in Fig 2D that the IC50 of Ru-SR1# and Ru-SR3# was relatively higher in HBE cells (both were higher than 30 μM) than in PANC 1, we indicated that Ru-SR1# and Ru-SR3# might serve as selective anti-pancreatic cancer drugs. For the sake of evaluating the radiosensitizing effects of Ru-SR1# and Ru-SR3# on pancreatic cancer cells, the concentration of 500 nmol was chosen for the following study, which elicited moderate inhibition with the cell viability around 80%~90% in PANC 1 cells.

**Figure 3:**
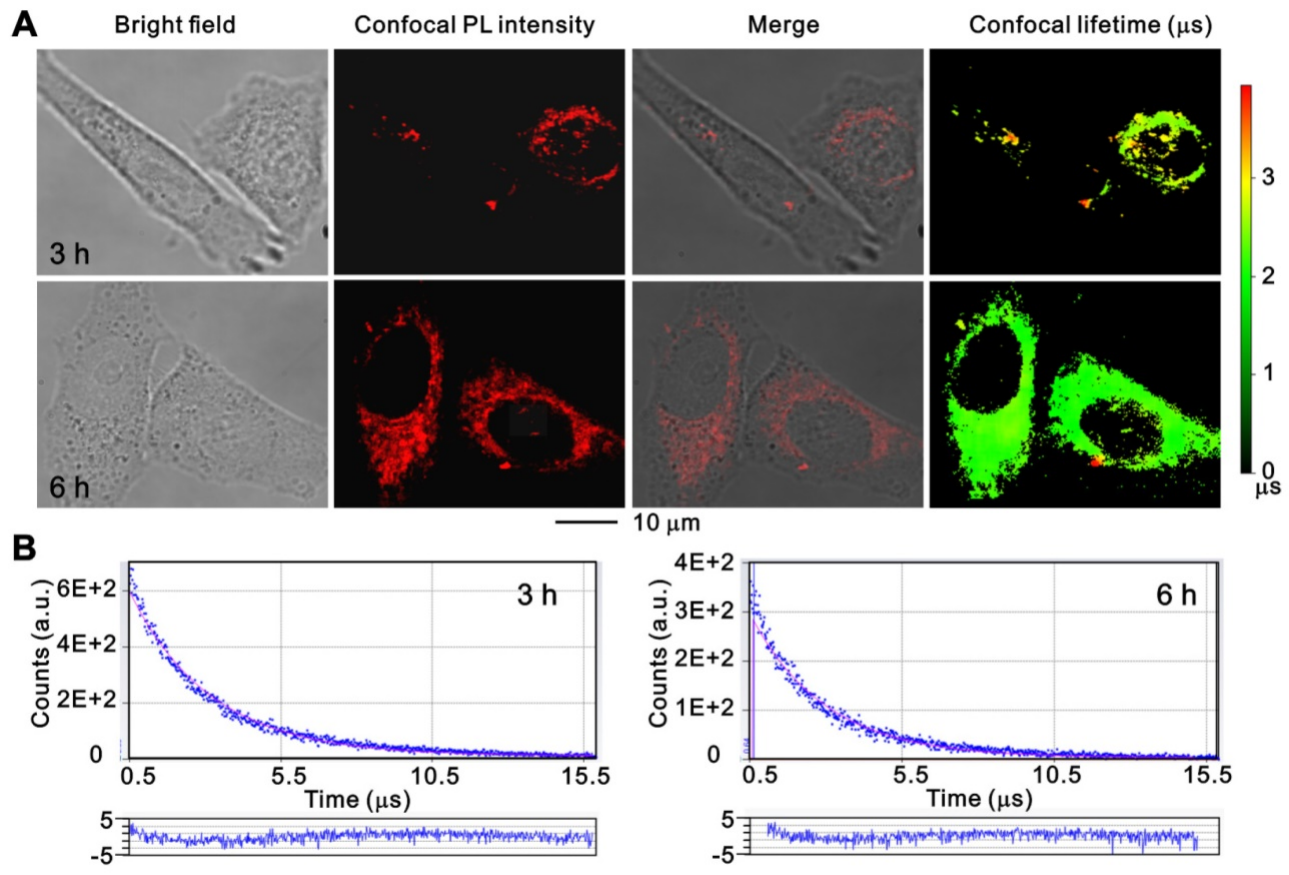
** The confocal images of PANC 1 cells after incubation with Ru-SR3# using time-resolved fluorescence microscopy.** PANC 1 cells were incubated with 500 nmol Ru-SR3# for 3 h and 6 h, and fixed by 4% paraformaldehyde. (**A**) The photoluminescence and lifetime images were observed and photographed using a confocal microscopy. (**B**) The emission decay curves and their fitting curves of Ru-SR3# in cells. The fitting curves were generated using single-exponential method and the residuals were plotted in the bottom panels.

To further investigate the cell uptake process of ruthenium(II) complex, PANC 1 cells were treated with Ru-SR3# for 3 h and 6 h and monitored using a time-resolved fluorescence microscope. As shown in Figure 3, the luminescent ruthenium(II) complex can rapidly enter the cell cytoplasm, and a small portion of molecule can further get into the nuclei at 3 h, resulting in a distinguishable observation of long-lifetime phosphorescence signals in nuclei (Fig 3, and Fig S11 in supplementary Materials). With prolongation of the incubation time to 6 h, the cell uptake of ruthenium(II) complex was increased, and the photoluminescence (PL) intensity of cell was elevated. The images were further co-stained with Mito-tracker Green to clarify the binding capability between mitochondrial and ruthenium(II) complex. As shown in Figure S11, the non-coincident luminescent signals suggest that the mitochondrial might not the main pathway related to ruthenium(II) complex caused cell death. It's notable that the long-lived luminescence signal should be mainly ascribed to endocytosed lipophilic ruthenium(II) complex, which can be easily differentiated from short-lived autofluorescence from cancer cells in the time domain, by using time-resolved fluorescence microscopy 27.

### Ru-SR3# radiosensitizes pancreatic cancer *in vitro*

**Figure 4:**
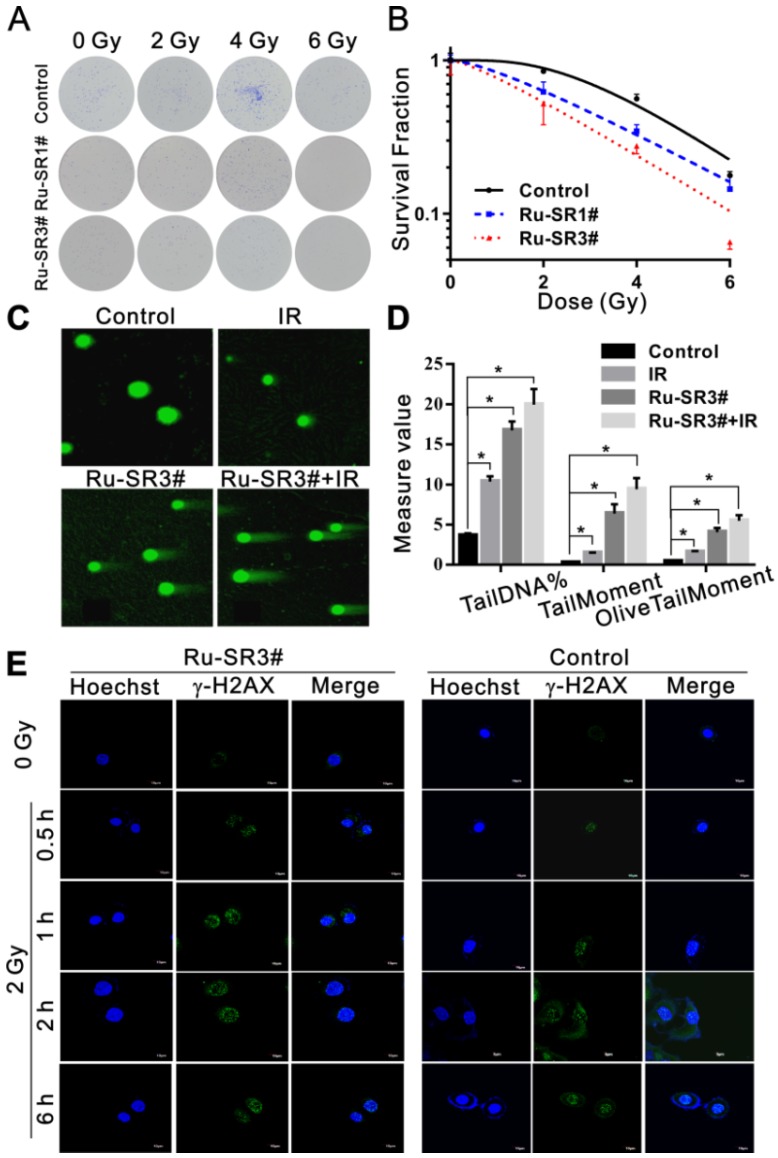
** Ruthenium complexes increased the radiosensitivity of pancreatic cancer cells.** (**A**) PANC 1 cells were treated with 500 nmol Ru-SR1# or Ru-SR3# for 24 h and then were exposed to 2, 4 or 6 Gy of X-ray. The cells were cultured at 37 °C for additional 10 d, and the number of colonies consisting of 50 or more cells was counted under a microscopy. (**B**) Clonogenic survival curve of PANC 1 cells treatment with Ru-SR1# or Ru-SR3# and X-ray irradiation were plotted and the values of D0, Dq, and SER were calculated using GraphPad Prism software (Table 2). (**C**) PANC 1 cells were treated with 500 nmol Ru-SR3#, 4 Gy X-ray, or Ru-SR3# plus X-ray. After 24 h, the cells were collected for DNA double strand breaks (DSBs) analysis using neutral single cell gel electrophoresis (SCGE). For each treatment, 100 cells were randomly chosen and photographed under a confocal microscopy. (**D**) The extent of DSBs in each treatment group was analyzed using Comet Assay Software Project (CASP), which was presented as the Tail DNA%, Tail Moment, and Olive Tail Moment. *, compared to control group, P<0.05. (**E**) After 0.5 h exposure to 2Gy X-Ray, the foci number of γ-H2AX in PANC 1 cells pretreated with 500 nmol Ru-SR3# was significantly more than that of the control group. All experiments mentioned above were performed at least three times.

The influence of Ru-SR1# and Ru-SR3# on the radiosensitivity of PANC 1 was determined via clonogenic assay. Cells were exposed to 500 nmol of Ru-SR1# and Ru-SR3# for 24 h, irradiated and respectively applied for clonogenic assays as described previously 28. As shown in Fig 4A and 4B, PANC 1 exposed to Ru-SR3# and X-ray presented lowest survival curve than the cells treated with Ru-SR1# and/or irradiation (IR) alone. In the clonogenic study, 500 nM Ru-SR in cancer cells was used for subsequent experiments. In radiobiology, the cell death refers to the loss of reproductive activity of the clonogenic cells, which generally occurs after the first or several divisions post-IR, and the appropriate observation time point is 9 to 14 days after IR exposure. The survival curves were generated by fitting survival fraction (SF) to the conventional “single-hit multi-target” model with the formula as SF=1-(1-e^-D/D0^)^N^ by GraphPad Prism software. According to the parameters of radiosensitization listed in Table 2, Ru-SR3# was further determined as a better candidate radiosensitizer for pancreatic cancer, which exhibited much higher sensitivity enhancement ratio (SER) than Ru-SR1# in PANC 1. In addition, it was found as expected that Ru-SR3# strengthened the IR-induced cell cycle G2/M phase arrest and apoptosis of PANC 1 cells (Figure S13A&S13B in Supplementary Material).

### Ru-SR3# enhances the radiation-induced DNA damage in PANC 1 nucleus

To explore the underlying mechanisms of Ru-SR3# in regulating radiosensitivity, PANC 1 cells pre-treated with/without 500 nM Ru-SR3# were exposed to 4 Gy X-ray and used to evaluate the induction of DNA double strand breaks (DSB) via neutral single cell gel electrophoresis. As shown in Fig 4C and 4D, Ru-SR3# pre-treatment and IR exposure could induce DSB in PANC 1 cells as compared to vehicle treated control cells, separately (P < 0.05); the combined treatment with Ru-SR3# and IR significantly increased the amount of “tail” formed by damaged DNA double-stranded debris, indicating enhanced DSBs as compared to control cells (P < 0.05). Furthermore, the immunofluorescence staining of radiation-induced phosphorylated histone γ-H2AX foci, the well-known DSB marker used in radiobiology, was performed (Figure 4E and Figure S12 in Supplementary Material). The number of foci was calculated from at least 100 cells per group, and the result indicated that Ru-SR3# pre-treated alone could cause significantly increased phosphorylated histone γ-H2AX foci formation, contrast to control cells (P < 0.05). When combined with 2 Gy X-ray exposure, obviously deteriorated DSB manifested in nucleus pre-treated with Ru-SR3#, as compared to that in X-ray exposed cells (P < 0.05).

**Figure 5:**
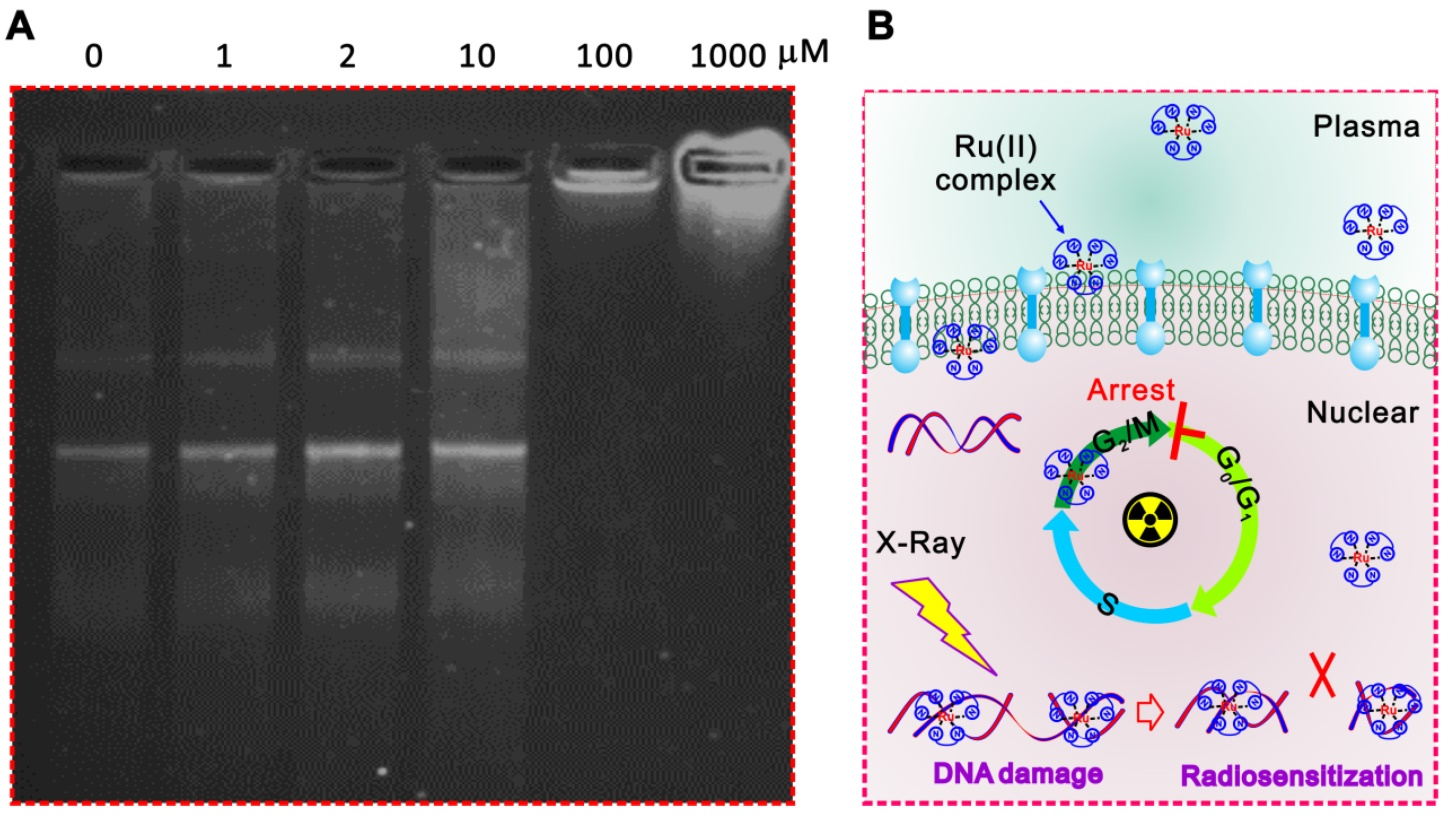
** The possible mechanisms related to the radiosensitizer capacity of ruthenium complexes.** (**A**) Ru-SR3# directly combined to DNA molecules. The recombinant plasmid DNA PGL3-LUC (2 µg) was incubated with 0, 1, 2, 10, 100, 1000 µmol Ru-SR3# for 1 h, then proceeded to 1% agarose gel electrophoresis at 8 V/cm voltage for 30 min. After staining by SYBR safe DNA gel stain reagent, the image was observed and captured by FluroChem M imaging system. (**B**) A schematic of ruthenium complex enhanced IR-induced DSB.

In addition, the significantly inhibited gel shifting was illustrated in Ru-SR3# pre-treated plasmid DNA via DNA agarose gel electrophoresis assay compared with CDDP, indicating the direct interaction between Ru-SR3# and DNA molecules (Figure 5A and Figure S14 in Supplementary Material). It's indicated that the Ru-SR3# has a stronger DNA binding affinity compared with CDDP, although the CDDP exhibits a higher DNA damage capability. Typically, IR-induced DNA single strand breaks (SSB) or double strand breaks (DSB) are often occur very shortly post-exposure (<10^-10^s), while the procedure of DNA repair is usually followed up within 10^2^s after exposure. As the outcome, cells will survive if the IR-induced DNA damage is successfully repaired. Otherwise, cells will undergo IR-induced cell death. During the processes above, DSBs firstly invokes phosphorylations of the histone variant H2AX, as well as the accumulation of several key factors mediating DNA damage signaling. In response to DSBs, phosphorylation of γ-H2AX seems to play a critical role, thus the employment of γ-H2AX has been extensively used to monitor the extent of DSB induction and analyze the effectiveness of novel biological therapies. In line with this finding, we found that Ru-SR3# enhanced a higher amount of γ-H2AX foci (Figure 4E and Figure S12 in supplementary material), which indicted potentially lethal DNA damage in Ru-SR-treated cancer cells post-radiation. IR-induced DSB will provoke a complex cellular response which activates and coordinates cell-cycle checkpoints, damage repair, and the eventual onset of apoptosis. Most mammalian cells exhibit transient delays in the G1 and G2 phases post-IR to allow the cell to correct possible defects. In our study, we demonstrated that Ru-SR treated PANC 1 cells which was exposed to IR led to an increase in the percentage of apoptotic cells, as compared with control cells (Figure S13B in supplementary material).

Taken together, the results above indicated that Ru-SR3# not only served as an excellent anti-pancreatic cancer chemotherapy reagent, but also acted as a valuable radiosensitizer for PANC 1 cells triggered by DNA damage response. Ru-SR3# alone can cause cycle G2/M phase arrest and apoptosis of PANC 1 cells; more interestingly, Ru-SR3# can profoundly strengthen the IR-induced PANC 1 cell G2/M phase arrest and apoptosis (Figure S13 in the supplementary materials). The mechanisms involved are thought to be related to the superior capability of ruthenium complexes for direct interaction with DNA molecules, which accounts for the enhanced IR-induced DSB (Figure 5B).

### Fluorescence imaging and radiotherapy of human pancreatic cancer xenograft nude mice

**Figure 6:**
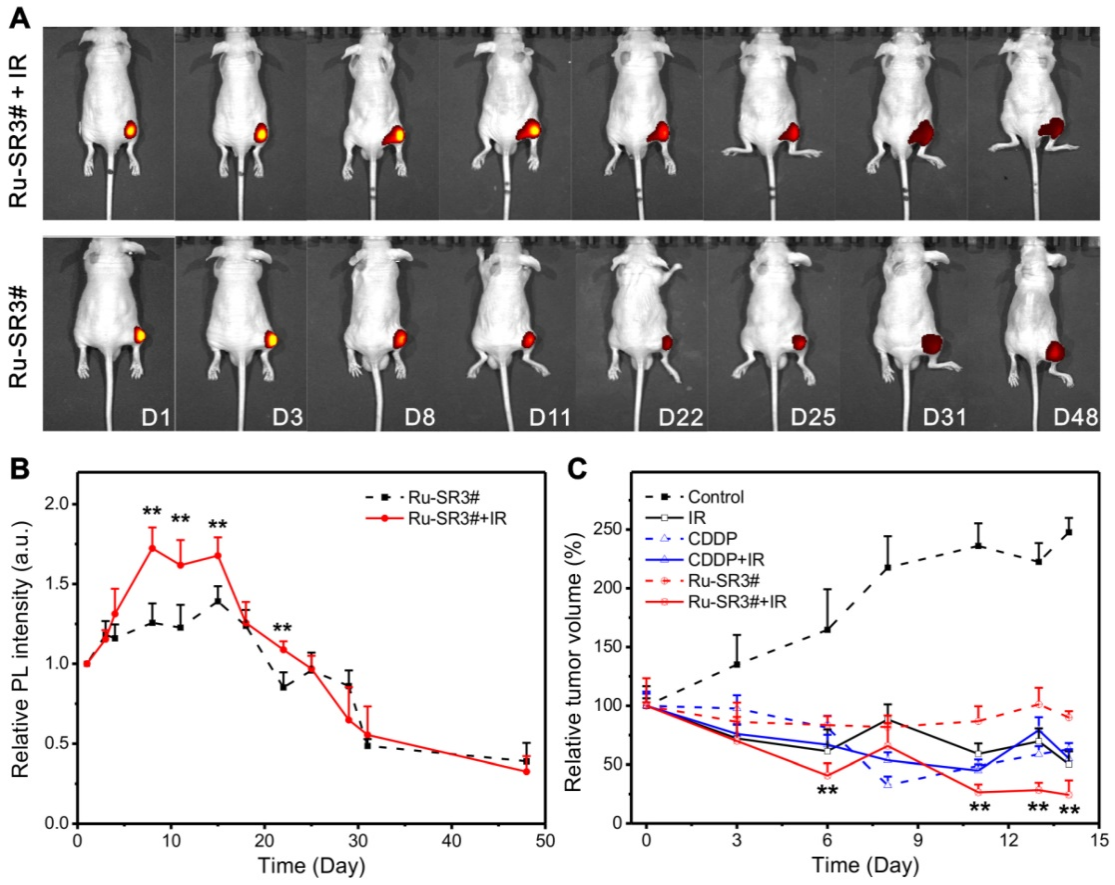
***In vivo* fluorescent imaging and validation for its radiosensitizer property of Ru-SR3# in human pancreatic cancer xenograft nude mice.** The human pancreatic cancer xenograft nude mice were constructed by intratumorally injecting 5×10^6^/0.1 mL PANC 1 cells into the right hind flank of nude mice. When the tumor volume reached 100 mm^3^, the mice was proceeded to different assays. (**A**) Photoluminescence images and (**B**) relative intensities (λ_ex_ 460 nm, λ_em_ 580-680 nm) of PANC 1 xenograft nude mice after i.d. injection with Ru-SR3# monitored at the different time post injection. (**C**) Tumor volume was measured every 2 days, which was calculated using formula *V* (mm^3^) = (*a×b^2^)/2*, where *a* is the length and *b* the width of the tumor tissue. **, compared to control group, P<0.01.

Based on the above data, the radiosensitizer properties of Ru-SR3# was further verified *in vivo* by applying human pancreatic cancer xenograft nude mice. Ru-SR3# was given to mice by intratumoral injection into mass at 20 mg/m^2^ (=8.3 mg/kg). CDDP was used as a positive control with the same treatment protocol in the present study. All PANC 1 xenograft mice were randomly separated into 6 groups (n=6): (1) intratumoral injection of DMSO (negative control); (2) intratumoral injection of CDDP (20 mg/m^2^); (3) intratumoral injection of Ru-SR3# (20 mg/m^2^); (4) The combination of DMSO and irradiation (20 Gy of electron beam); (5) The combination of CDDP and irradiation; (6) The combination of Ru-SR3# and irradiation. The luminescence of ruthenium(II) complex in mice was quantitatively analyzed by using small animal fluorescence imaging system. As illustrated in Fig 6A and 6B, the Ru-SR3# injections generate an observable luminescence with an excitation of 420-460 nm light. Notably, 4-6 days after cell injection, luminescence intensity seemed to reach its peak value mainly because the medium was absorbed by the body leading to the transiently elevated concentration of ruthenium(II) complex. Afterwards, the visible decrease of luminescence intensity can be found after peak value, which might be ascribed to the metabolization of Ru-SR3#. Taken together, these results indicated the ruthenium complex can emit visible luminescence during the tumor treatment procedure.

It was also indicated that the Ru-SR3#, CDDP or IR treatment alone manifested the similar suppression effects in contrast to sham-treated control group in PANC 1 xenograft mice. As shown in Fig 6C, IR, CDDP or Ru-SR3# treatment respectively reduced the tumor volume by 50%, 47% or 10%, when compared with the control group at day 14 after treatment. Comparatively, the combined treatment of IR exposure and Ru-SR3# showed more effective suppression on PANC1 xenografts growth as compared with CDDP plus IR treatment (tumor volume reduced by 75 % in IR plus Ru-SR3# group v.s. 45% in IR plus CDDP group). In the meanwhile, the body weight of Ru-SR3# treated mice has no significant change compared with control groups (Figure S15 in supplementary material). Moreover, the effect of IR and/or Ru-SR3# treatment on human pancreatic cancer xenografts was further investigated by hematoxylin-eosin (H&E) staining and immunohistochemistry (IHC) staining of ki67 expression (Figure S16 in supplementary material).

It's found that the serious necrosis and hemorrhage were observed in xenografts treated with the Ru-SR3 plus IR exposure, as compared to the other treatment groups. The ki67 expression in CDDP and Ru-SR3# plus IR group was almost depleted, indicating a neglectable cell proliferation. These results were consistent with the *in vitro* data described above.

## Conclusions

In summary, we synthesized ruthenium(II) complexes with variation number of DIP ligand and further evaluated their potential as radiosensitizer for pancreatic cancer radiotherapy. The long-lifetime ruthenium(II) complexes can get into the cell nuclei and directly combine with DNA, which contribute to the enhanced cell death by aggravating IR-induced DSB. The luminescence of ruthenium(II) complex can be adopted as a guiding indication during cancer radiotherapy, and ruthenium(II) complex combined with IR can effectively suppress pancreatic tumor growth. Therefore, our study provides novel and promising ruthenium(II) polypyridyl complexes which is likely to benefit the imaging-guided cancer radio-chemotherapy.

## Experimental section

### Materials and methods

DIP, 2,2-bipyridine, ruthenium(III) chloride hydrate and *cis*-bis-(2,2'-bipyridine)dichlororuthenium(II) dihydrate were all purchased from J&K Chemical Ltd. Dichloromethane, *n*-hexane and methanol were all obtained from Sinopharm Chemical Reagent Co. Ltd. NMR spectra were acquired on a VARIAN 400 M magnetic resonance spectrophotometer. ^1^H NMR and ^13^C NMR spectra were acquired on a VARIAN 400 MHz magnetic resonance spectrophotometer. The solvent signals were used for ^1^H NMR (δ(DMSO-d_6_) = 2.50 ppm) and ^13^C NMR (δ(DMSO-d_6_) = 39.5 ppm) as the internal standard. Mass spectra were measured on a Varian ProStar LC240 (America). UV-vis spectra, PL spectra and emission lifetime (τ_p_) were recorded on a UV-vis spectrophotometer (TU-1950, Beijing Purkinje General Instrument Co., Ltd, Beijing, China) and an Edinburgh FLS920 type steady-state/ transient spectrometer, respectively. Photoluminescence images and emission lifetime measurements were performed using a time-resolved spectroscopy system (ISS Q2 with FastFLIM) based on a pulsed laser and a fast-gated detector. The excitation light was set up to 460 nm. The emission spectra were collected from 580 nm to 700 nm. The exit port of the spectrometer was connected to a time-gated image with a fast acquisition gate adjustable from 50 ps to continuous mode.

### Synthesis

**Ru-SR1#:** The mixture of cis-bis-(2,2'-bipyridine)dichlororuthenium(II) dihydrate (187 mg, 0.36 mmol), 4,7-diphenyl-1,10-phenanthroline (DIP, 120 mg, 0.36 mmol) and methanol (20 mL) are stirred and heated at the boiling temperature under argon atmosphere for 12 hours. After cooling to room temperature, the solvent is removed by rotavapor. The crude product was purified by column chromatography on alumina using dichloromethane-methanol (v:v=2:1) was the eluent. Yield: 76%. ^1^H NMR (400 MHz, DMSO-d_6_), δ (ppm): 8.98-8.84(m, 2 H), 8.30-8.10 (m, 4 H), 7.87 (t, J=5.6 Hz, 2 H), 7.74-7.58 (m, 7 H), 7.46 (t, J=6.8 Hz, 1 H). ^13^C NMR (101 MHz, DMSO-d_6_), δ (ppm): 157.27, 157.05, 152.29, 152.10, 151.80, 148.37, 148.13, 138.51, 138.43, 135.85, 130.38, 130.13, 129.61, 129.28, 129.12, 128.56, 128.42, 127.02, 126.51, 125.04. Tof-MS (M-2Cl)^2+^, m/z: 370.0857(observed); 370.0877 (calculated), UPLC purity: 99.3%.

**Ru-SR2#:** Step 1. The mixture of ruthenium(III) chloride hydrate (655 mg, 2.5 mmol), DIP (1.7 g, 5.1 mmol) and anhydrous LiCl (588 mg, 14 mmol) are dissolved in DMF (10 mL) and refluxed for 12 hours under argon atmosphere. After cooling to room temperature, 50 mL acetone was added to the above mixture solution. After stirring for 5 minutes, the mixture solution was kept in refrigerator at -20 °C for overnight. Then, the precipitate is filtrated and washed with deionized water and diethyl ether. After drying under vacuum, the intermediate product of *cis*-bis-(4,7-diphenyl-1,10-phenanthroline) dichlororuthenium(II) dihydrate (abbreviated as *cis*-Ru(DIP)_2_Cl_2_, 1.6 g) was obtained without further purification for the next step. Yield 53%. Step 2. The mixture of *cis*-Ru(DIP)_2_Cl_2_ (250 mg, 0.3 mmol) and 2,2'-bipyridine (bpy, 50 mg, 0.32 mmol) are dissolved in methanol and refluxed for 12 hours at argon atmosphere. After cooling to room temperature, the solvent is removed by rotary evaporation. The crude product was purified by column chromatography on alumina using dichloromethane-methanol (v:v=2:1) was the eluent. Yield: 87%. ^1^H NMR (400 MHz, DMSO-d_6_), δ (ppm): 8.96 (d, J=8 Hz, 1 H), 8.33 (d, J=5.6 Hz, 1 H), 8.26-8.23 (m, 4 H), 7.95 (d, J=5.2 Hz, 1 H), 7.87 (d, 5.2 Hz, 1 H), 7.77-7.54 (m, 12 H). ^13^C NMR (DMSO-d_6_, 101 MHz) δ (ppm): 157.33, 152.77, 152.44, 152.24, 148.44, 148.42, 148.36, 148.23, 138.57, 135.90, 135.86, 130.42, 130.38, 130.14, 130.11, 129.64, 129.60, 128.60, 128.47, 127.08, 127.01, 126.55, 126.48, 125.11. Tof-MS (M-2Cl)^2+^, m/z: 458.1189 (observed); 458.1190 (calculated), UPLC purity: 98.2%.

**Ru-SR3#:** This complex was synthesized using the same procedure as that for Ru-SR2#, with 4,7-diphenyl-1,10-phenanthroline (DIP, 106 mg, 0.32 mmol) in the place of bpy in the Step 2. Yield: 68%.^1^H NMR (400 MHz, DMSO-d_6_), δ (ppm): 9.18 (d, J=4.4 Hz, 1 H), 8.37 (d, J=5.6 Hz, 2 H), 8.29 (s, 1 H), 7.86 (m, 3 H), 7.75-7.55 (m, 16 H). ^13^C NMR (101 MHz, DMSO-d_6_), δ (ppm): 152.88, 150.13, 148.52, 148.43, 137.77, 135.88, 130.40, 130.10, 129.66, 129.29, 129.14, 128.61, 127.02, 126.13, 124.36, 124.16. Tof-MS (M-2Cl)^2+^, m/z: 546.1522(observed); 546.1503 (calculated), UPLC purity: 98.4%.

### Cell culture

Human pancreatic cancer cell line PANC1, esophageal cancer cell line TE-1, lung cancer cell line H1299, and normal pulmonary bronchial cell line HBE were purchased from Procell Life Science & Technology Co. Ltd. (Wuhan, China). Cells were grown in DMEM medium, supplemented with 10% FBS, 100 U/ml penicillin and 100 U/ml streptomycin (Invitrogen, Carlsbad, CA, USA), and cultured at 37℃ in a humid atmosphere containing 5% CO_2_.

### Cell viability assay

Exponential cells were seeded in 96-well plates at density of 5×10^3^/ml, incubated overnight, then were treated with different concentrations of Ru-SR3# for indicated days. Cell viability was detected using Cell Counting Kit-8 (CCK-8) (DOJINDO Laboratories, Japan). Half maximal inhibitory concentration (IC_50_) was calculated by the algebraic formula generated via regression analysis by SPSS19.0.

### Colonogenic Assay

After treated with 500 nmol of Ru-SR1# and Ru-SR3# for 12h, cells were separately seeded in 6-well plates at different densities (200, 200, 400, 800, and 1600 cells for 0Gy, 2Gy, 4Gy, 6Gy and 8Gy), and respectively exposed to 0, 2, 4, 6, 8Gy X-ray generated by an X-ray linear accelerator (dose rate of 1.15 Gy/min, RadSource, Suwanee, GA, USA). Ten to 14 days of incubation in 5% CO_2_ at 37°C, cells were rinsed with PBS for 2 times, fixed in methanol followed by Giemsa staining. The number of colonies consisting more than 50 cells were counted, and the surviving fraction was calculated and fitted into the linear quadratic model as described previously 28.

### Flow cytometry analysis

Cell apoptosis and cell cycle analysis were performed using flow cytometry assay as described previously 28. In brief, for apoptosis analysis, cells were collected and stained with 10 µg/ml propidium iodide (PI, Beyotime Biotechnology, Haimen, China) for 1 h and Annexin V (Beyotime Biotechnology, Haimen, China) for 15min and proceeded to flow cytometry analysis. For cell cycle distribution analysis, cells were collected and washed with PBS, fixed with pre-chilled 70% ethyl alcohol at 4 °C for 4 h. After washed with PBS, cells were incubated with 500 µL PBS containing 50 µL of a 100 µg/mL sock of RNase A and PI (50 µg/mL stock solution) at 37 °C in dark for 30 min. The apoptotic rate and cell cycle distribution were calculated from 10000 cells using ModFit LT software (Becton Dickinson, CA, USA) using FACS Calibur (Becton Dickinson, San Jose, CA, USA).

### Immunofluorescence staining assay

Cells were seeded in glass bottom plate (Corning, NY, USA). After corresponding treatment, cells were fixed with 4% paraformaldehyde for 30 min at room temperature, washed with PBS for 3 times, and permeabilized with 0.2% Triton X-100 for 15 min at room temperature. After blocked in 1% BSA solution at room temperature for 1 h, cell were incubated with anti-phosphorylated γ-H2AX (1:2000, Cell Signaling Technology, Boston, MA, USA) overnight at 4 °C. Cells were washed and incubated in appropriate secondary antibody conjugated with FITC (1:1000, Beyotime Biotechnology) for 1 h at room temperature in the dark. Then nuclei were visualized by 2-(4-Amidinophenyl)-6-indolecarbamidine dihydrochloride (DAPI, Beyotime Biotechnology, Haimen, China) staining and images were acquired by a confocal microscope (Olympus, Tokyo, Japan).

### Single cell gel electrophoresis

DNA damage was analyzed using Trevigen Comet Assay^®^ Kit (Gaithersburg, MD, USA) according to the manufacturer's instructions. Briefly, 1 x 10^5^/ml cells were re-suspended with molten LMAgarose (at 37 °C) and immediately loaded onto CometSlide™. Slides were immersed in Lysis Solution at 4 °C for 60 min, followed by electrophoresis in 1× neutral electrophoresis buffer with a voltage at 1 volt/cm. The diluted SYBR® Green was applied for DNA staining. The images were captured using epifluorescence microscopy (Olympus, Tokyo, Japan), and the DNA damages parameters were assayed using the free Comet Assay Software Project.

### *In vivo* fluorescent imaging and radiotherapy of human pancreatic cancer xenograft mice

Female BALB/C nude mice were purchased from SLAC Laboratory Animal Co., Ltd. (Shanghai, China), and maintained in a pressurized ventilated cage at 25 ℃ with a 12-h dark/light cycle according to institutional regulations. PANC 1 cells (5×10^6^) were intratumorally injected into the right hind flank of nude mice. When the tumor volume reached 100 mm^3^, the mice was randomly separated into 6 groups (n=6): (1) intratumoral injection of DMSO (negative control, every other day throughout the experiment); (2) intratumoral injection of *cis-*diaminodichloroplatinum (CDDP, 20 mg/m^2^, every other days throughout the experiment); (3) intratumoral injection of Ru-SR3# (20 mg/m^2^, every other days throughout the experiment); (4) The combination of DMSO and irradiation; (5) The combination of CDDP and irradiation; (6) The combination of Ru-SR3# and irradiation. Tumor volume was measured every 2 days, which was calculated using formula *V* (mm^3^) = (*a×b^2^)/2*, where *a* is the length and *b* is the width of the tumor tissue.

Tumors were exposed to 20 Gy of electron beam irradiation by a 6 MeV X-ray linear accelerator (KD-2, Siemens, German) at a dose rate of 200 cGy/min 29.At the end of the treatment, the animals were euthanasia sacrificed. All animal experiments were approved and conducted according to the Research Ethics Committee at Soochow University, and in line with the guide for the Care and Use of Laboratory Animals.

### DNA electrophoresis

The human Nrf2 promoter recombinant plasmid pLG3-Nrf2 was dissolved in buffer containing 10 mM Tris and 1mM EDTA (pH 7.4). The same amount plasmid was separately incubated with different concentrations of Ru-SR3# for 1 h, then was loaded onto 1% agarose gel. After electrophoresis under 120 voltage for 45 min, the plasmid DNA was visualized as clear bands against the background, and photographed using a FluroChem M imaging system (Proteinsimple, San Jose, CA).

### Statistical analysis

All data were presented as the means ± standard error of the mean (SEM) of three independent experiments. One way ANOVA was performed to determine the statistical significance of differences. Prism 6 software (GraphPad) as utilized for the statistical analyses. P < 0.05 was considered significant.

## Figures and Tables

**Table 1:** IC_50_ values of PANC 1, TE-1, H1299 and HBE cell lines treated with Ru complexes in Figure 2

	Complexes	PANC-1	TE-1	H1299	HBE
IC_50_ (µM)	Ru-SR1#	13.7	173.1	12	42.8
	Ru-SR2#	20.3	14.6	6.4	13.1
	Ru-SR3#	6.6	12.8	5	69.8

**Table 2:** The D_0_, D_q_, D_37_, N and SER values in control, Ru-SR1# and Ru-SR3# treated PANC 1 cells. The SER value was simulated using the multi-target single hit model.

Groups	D_0_	D_q_	D_37_	N	SER
Control	2.05	3.14	5.19	4.63	
Ru-SR1#	2.39	1.56	3.95	1.92	2.01
Ru-SR3#	2.42	0.69	3.11	1.33	4.54

## Abbreviations

en: ethylenediamine
PDT: photodynamic therapy
DIP: 4,7-diphenyl-1,10-phenoline
cis-Ru(bpy)2Cl2: cis-bis-(2,2'-bipyridine)dichlororuthenium (II) dihydrate
cis-Ru(DIP)2Cl2: cis-bis-(4,7-diphenyl-1,10-phenanthroline)dichlororuthenium(II) dihydrate
bpy: 4,4'-bipyridine
PL: photoluminescence
SER: sensitivity enhancement ratio
DSB: double strand breaks
DFT: density functional theory
TD-DFT: time-dependent density functional theory
CCK-8: cell counting kit-8
CDDP: cis-diaminodichloroplatinum
PI: propidium iodide
FITC: fluoresceine isothiocyanate
DAPI: 2-(4-Amidinophenyl)-6-indolecarbamidine dihydrochloride
EDTA: ethylene diamine tetraacetic acid
