# Supplementary material for: Luminescent ruthenium(II) polypyridyl complexes acted as radiosensitizer for pancreatic cancer by enhancing radiation-induced DNA damage
Source: Theranostics. 2019 Sep 18;9(22):6665–75. doi: 10.7150/thno.34015 (PMC6771246; doi:10.7150/thno.34015)
Supplement: Supplementary file 1 — Supplementary figures and tables. [file thnov09p6665s1.pdf]

Supplementary Material

Luminescent Ruthenium(II) Polypyridyl Complexes Acted as Radiosensitizer for Pancreatic Cancer by Enhancing Radiation-induced DNA damage

Yuyang Zhou[1,*], Ying Xu[2], Lunjie Lu[3], Jingyang Ni[1], Jihua Nie[2], Jianping Cao[2], Yang Jiao[2,*], Qi Zhang [2,*]

1. School of Chemistry, Biology and Materials Engineering, Jiangsu Key Laboratory of Environmental Functional Materials, Suzhou University of Science and Technology, Suzhou, Jiangsu, 215009, P. R. China.

2. School of Radiation Medicine and Protection, State Key Laboratory of Radiation Medicine and Protection, Medical College of Soochow University, Suzhou, Jiangsu 215123, P. R. China.

3. Department of Radiation Physics, Qingdao Central Hospital, Qingdao, Shandong, 266000, P. R. China.

* Corresponding author: Prof. Yuyang Zhou, E-mail: zhouyuyang@mail.usts.edu.cn. Prof. Yang Jiao, Tel: +86 0512-65883941, E-mail: jiaoyang@suda.edu.cn. Prof. Qi Zhang, Tel: +86 0512-65883941, Email: qzhang2012@suda.edu.cn

© The author(s). This is an open access article distributed under the terms of the Creative Commons Attribution License (https://creativecommons.org/licenses/by/4.0/). See http://ivyspring.com/terms for full terms and conditions.

1. The NMR and mass spectra of ruthenium(II) complexes

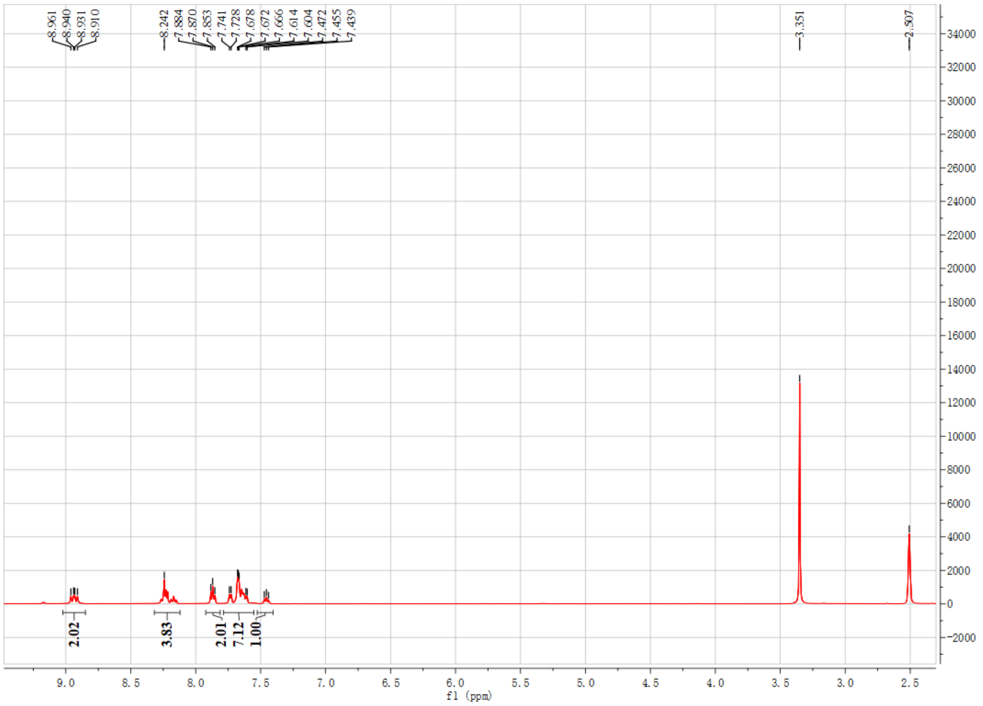

Figure S1: The ^{1}H NMR spectra of Ru-SR1# in DMSO-d_6.

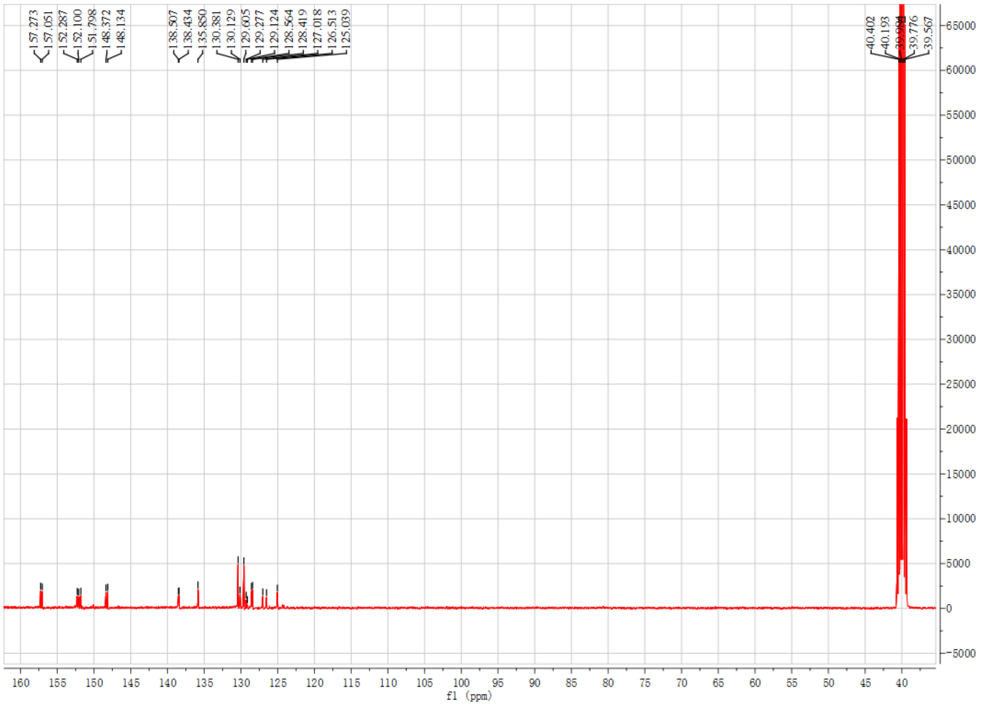

Figure S2: The ^{13}C NMR spectra of Ru-SR1# in DMSO-d_6.

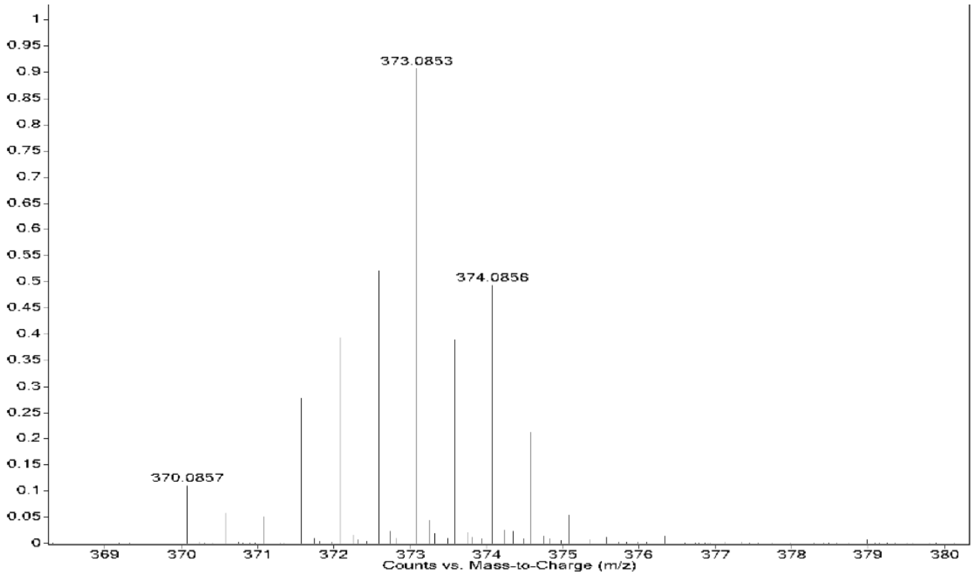

Figure S3: The mass spectra of Ru-SR1#.

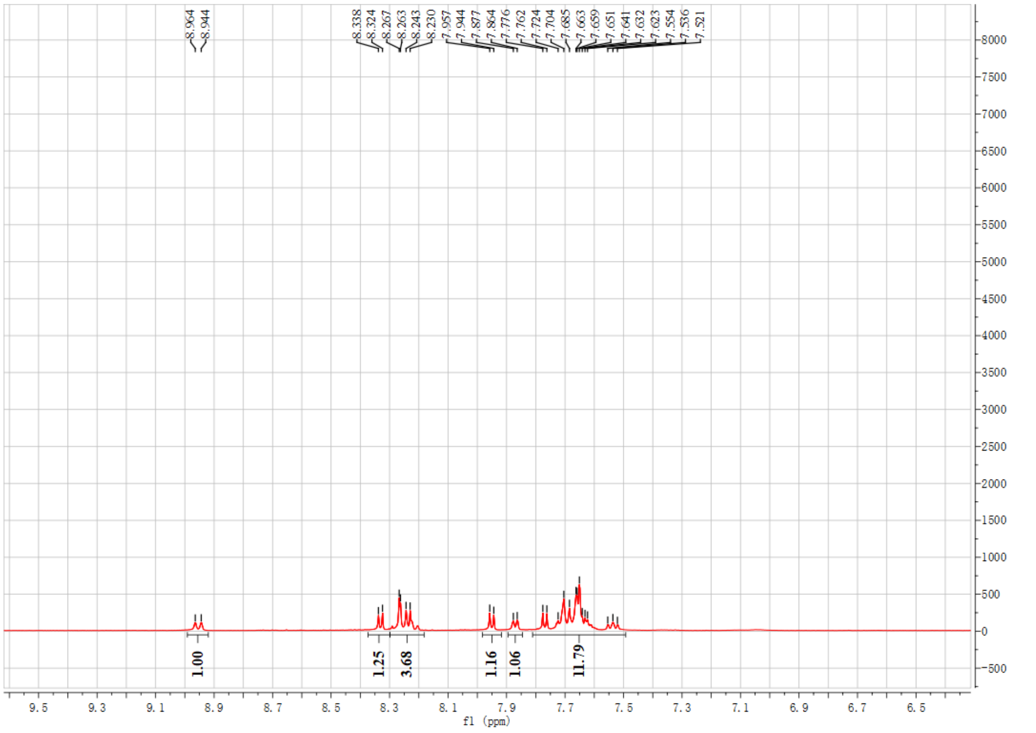

Figure S4: The ^{1}H NMR spectra of Ru-SR2# in DMSO-d_6.

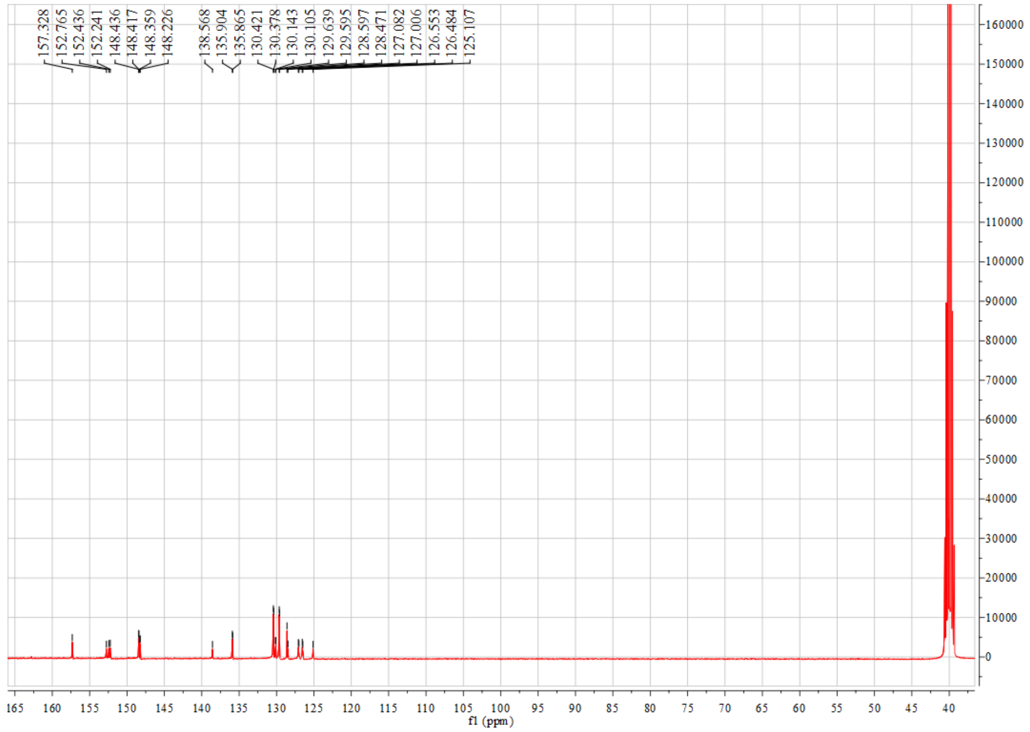

Figure S5: The ^{13}C NMR spectra of Ru-SR2# in DMSO-d_6.

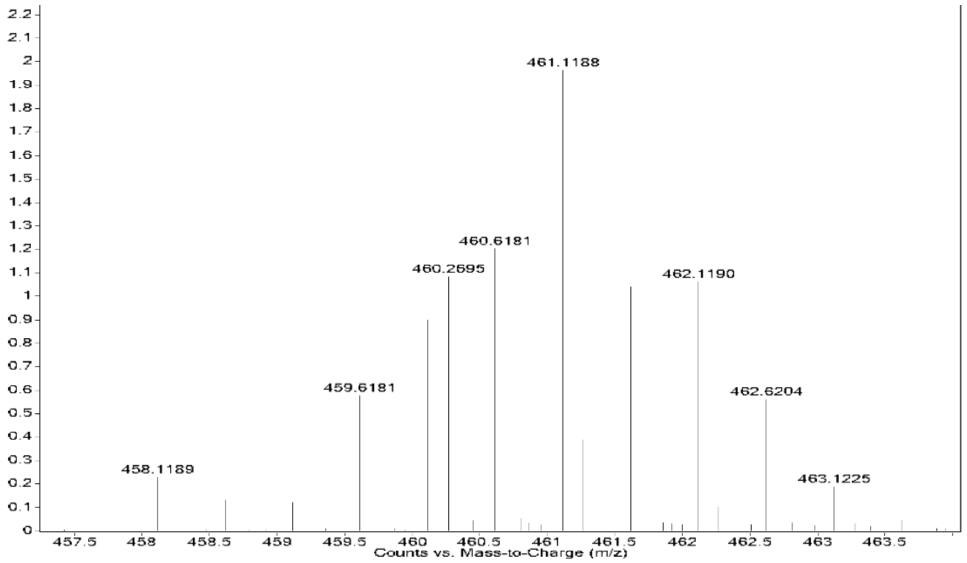

Figure S6: The mass spectra of Ru-SR2#.

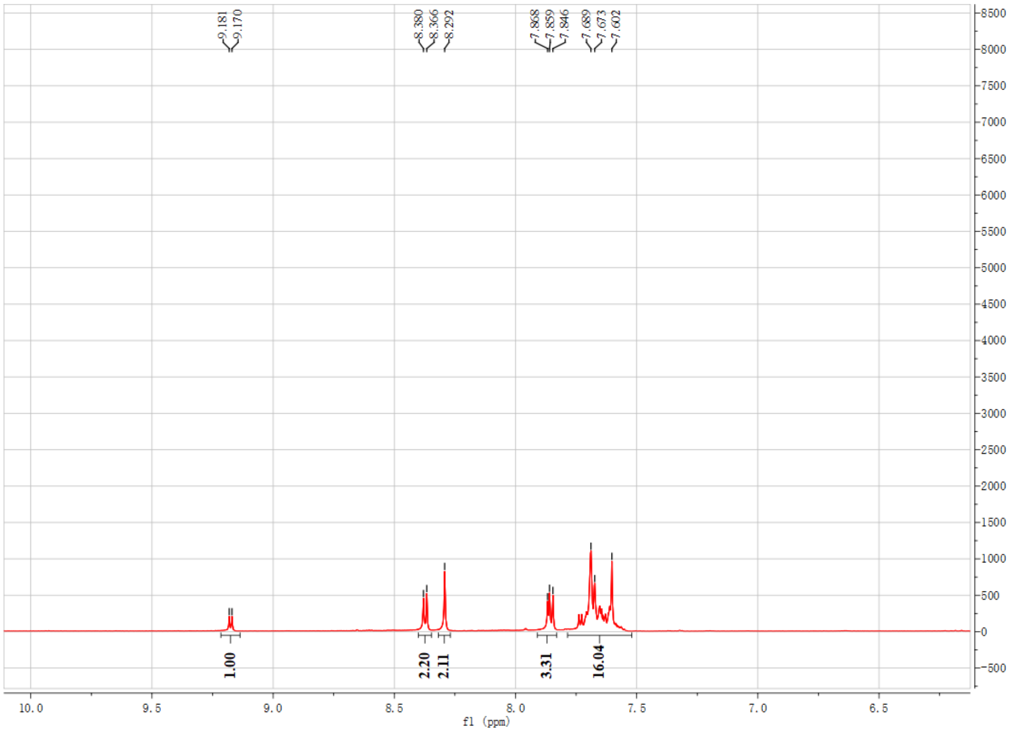

Figure S7: The ^{1}H NMR spectra of Ru-SR3# in DMSO-d_6.

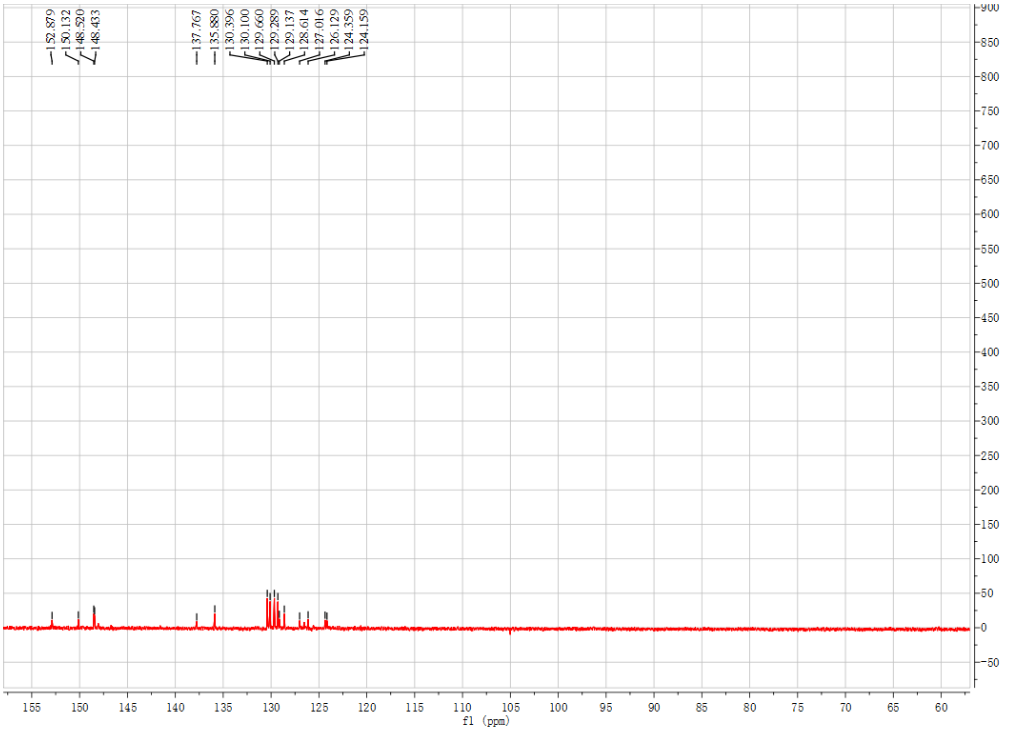

Figure S8: The ^{13}C NMR spectra of Ru-SR3# in DMSO-d_6.

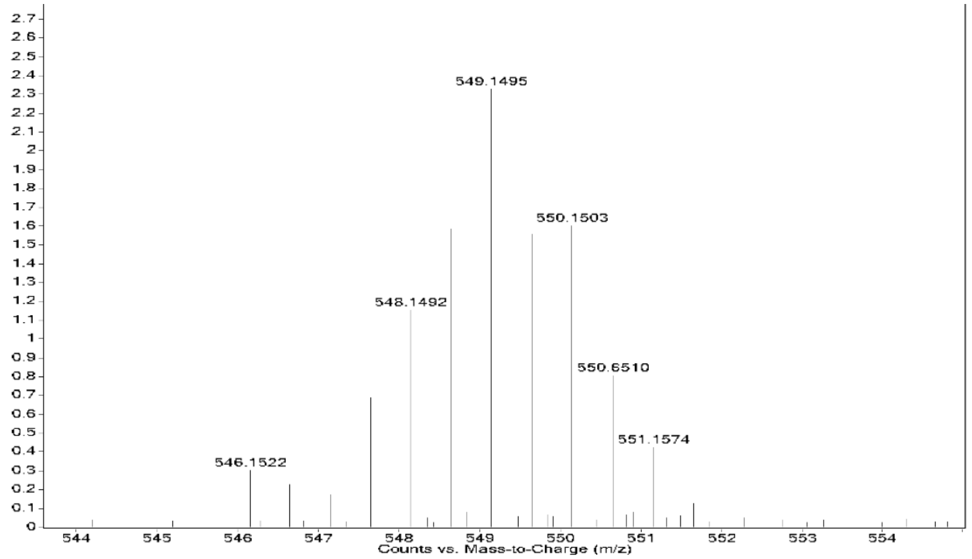

Figure S9: The mass spectra of Ru-SR3#.

2. Photophysical properties of ruthenium(II) complexes

Because the photophysical properties are important for the following series of in vitro and in vivo studies, the absorption and photoluminescent spectra were firstly characterized and listed in Figure S1. According to Figure S10A, these ruthenium(II) complexes in this work have moderate absorption intensity between 350 and 500 nm (the maximum ε in this region is approximate 12500 $M^{-1}cm^{-1}$) which was ascribed to MLCT state and comparatively strong absorption intensity below 350 nm (the maximum ε in this region is up to 60000 $M^{-1}cm^{-1}$) which was ascribed to intra ligand absorption band. Moreover, the major difference in absorption spectra was illustrated in the intra ligand absorption band when increasing DIP number from Ru-SR1# to Ru-SR2#. However, further increasing DIP number from Ru-SR2# to Ru-SR3# induced almost no difference in the whole regions of their absorption spectra. The photoluminescent (PL) spectra of these ruthenium(II) complexes as presented in Figure S10B are almost identical to each other which may be reasonably ascribed to the same MLCT states just as Figure S10A.

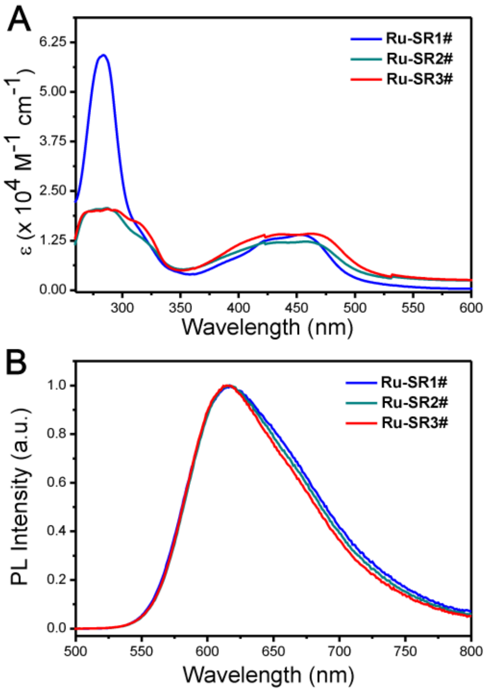

Figure S10: The absorption (**A**) and PL (**B**) spectra of luminescent ruthenium(II) complexes in methanol solution. ε the molar absorption coefficient. The concentration of ruthenium(II) complex is 40 μM, λ_{ex}=410 nm.

Table S1: Spectroscopic data of luminescent ruthenium(II) complexes in methanol

Complex	Absorption	Emission [a]			Log $P_{o/w}$
	λ (nm) [ε×10^{-4} $M^{-1}cm^{-1}$]	λ_{max} (nm)	Φ_{PL}	τ_p (μs)	
Ru-SR1#	283[5.93]; 422[1.24]<br>429[1.29]; 457[1.40]	617	0.063	5.78	-0.15
Ru-SR2#	278[2.04]; 286[2.08]<br>295[1.97]; 312[1.73]<br>433[1.22]; 460[1.23]	616	0.042	5.68	0.74
Ru-SR3#	271[1.99]; 287[2.04]<br>295[2.02]; 314[1.69]<br>435[1.43]; 465[1.44]	614	0.036	6.61	0.91

[a] deaerated condition, at room temperature. $Ru(bpy)_3$ (Φ_{PL}=0.062) was used as the reference for calculating quantum efficiency.

3. Theoretical analysis of ruthenium(II) complexes

In order to further understanding the photophysical properties, DFT and TD-DFT theoretical calculations (Gaussian 09 package) were also employed to investigated the ground and excited states of these ruthenium(II) complexes in this work. The electron distributions and energy level on frontier orbitals of these ruthenium(II) complexes are shown in Table S2. Obviously, along with increasing with DIP ligand, both HOMO and LUMO of ruthenium(II) complexes raised up from Ru-SR1#, Ru-SR2# to Ru-SR3#. According to Table S3, slight differences could be found among the energies of the optimized excited states (T_1 and S_1), which may theoretically explain the phenomenon of the almost identical PL spectra of these ruthenium(II) complexes as shown in Fig S1B.

Table S2: The electron distributions and energy levels of frontier orbitals based on the optimized ground state of luminescent ruthenium(II) complexes by DFT calculations

Complex	HOMO-1	HOMO	LUMO	LUMO+1
Ru-SR1#	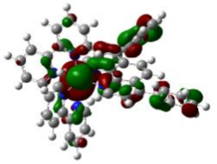<br>-10.51 eV	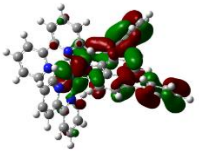<br>-10.43 eV	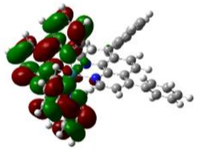<br>-7.18 eV	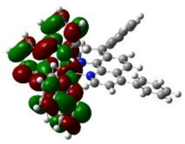<br>-7.10 eV
Ru- SR2#	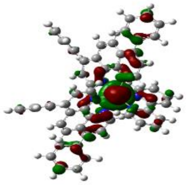<br>-10.15 eV	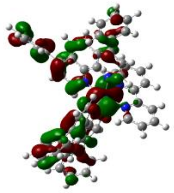<br>-10.09 eV	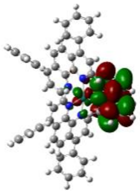<br>-6.88 eV	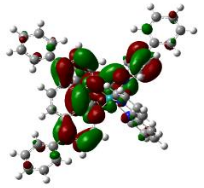<br>-6.64 eV
Ru- SR3#	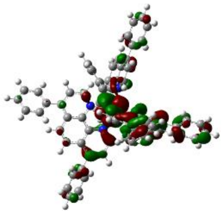<br>-9.83 eV	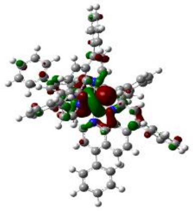<br>-9.83 eV	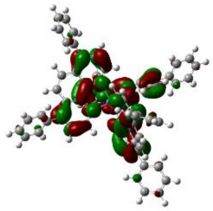<br>-6.46 eV	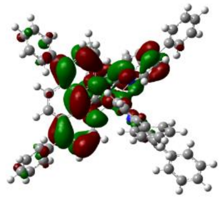<br>-6.35 eV

Table S3: TD-DFT calculation results about ruthenium(II) complexes

Complex	State	Energy (eV)	λ (nm)	*f*	Major assignments
Ru-SR1#	T_1	2.30 eV	538	0.0000	HOMO-1→LUMO+1 (0.62871)
	S_1	2.42 eV	513	0.0003	HOMO-1→LUMO+1 (0.67721)
Ru-SR2#	T_1	2.23 eV	556	0.0000	HOMO-1→LUMO (0.63149)
	S_1	2.37 eV	522	0.0010	HOMO-1→LUMO (0.65507)
Ru-SR3#	T_1	2.31 eV	538	0.0000	HOMO→LUMO+1 (0.49962)
	S_1	2.41 eV	514	0.0062	HOMO→LUMO (0.67944)

4. The cell behaviors of PANC 1

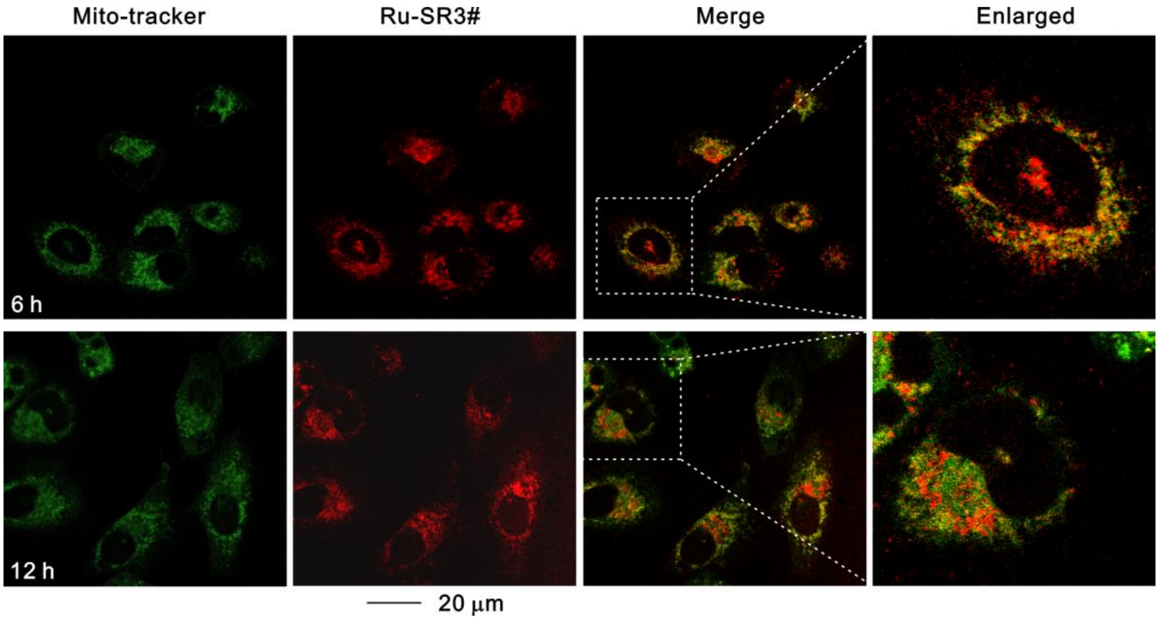

Figure S11: The images of PANC 1 cells cultured with Ru-SR3# using fluorescence confocal microscopy. To determine the cellular localization of Ru-SR3# in PANC 1 cells, cells were incubated with 500 nmol Ru-SR3# for 6h and 12h, respectively. At 30 min prior to fluorescence detection, 20 nM Mito-Tracker Green (Beyotime Corp) was added to the medium. The cells were then rinsed twice with 1 × PBS, fixed by 4% paraformaldehyde, and proceeded to fluorescence visualization using confocal microscopy analysis.

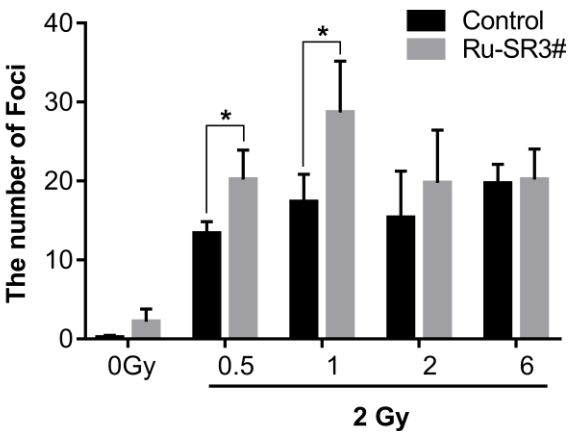

Figure S12: Ru-SR3# increased γ-H2AX foci induced by 2 Gy X-ray irradiation. The number of foci was counted from at least 100 cells per group. The PANC 1 cells were pretreated with 500 nmol Ru-SR3#, and the images were monitored at the indicated time after irradiation. *P<0.05.

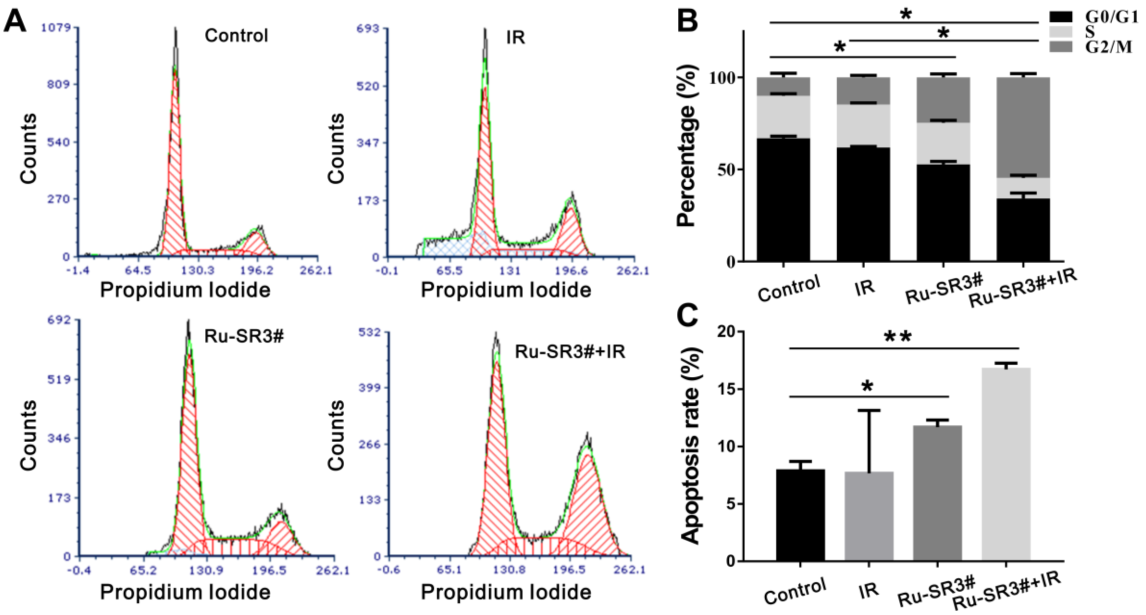

Figure S13: Cells were pretreated with/without 500 nmol Ru-SR3# for 24h, then were exposed to 4Gy X-rays. Cell cycle distribution and apoptosis were analyzed using flow cytometry assay. (**A-B**) Ru-RS3# enhances radiation-induced cell G2/M phase arrest. The ratio of G2/M phase in PANC1 cells pretreated with Ru-SR3# was significantly higher than that in untreated control group, as well as that in IR-exposure group. (**C**) Quantification of apoptosis percentage was measured by using Annexin-V/PI staining. Ru-RS3# can increase IR-induced apoptosis in PANC1 cells. *P<0.05, **P<0.01.

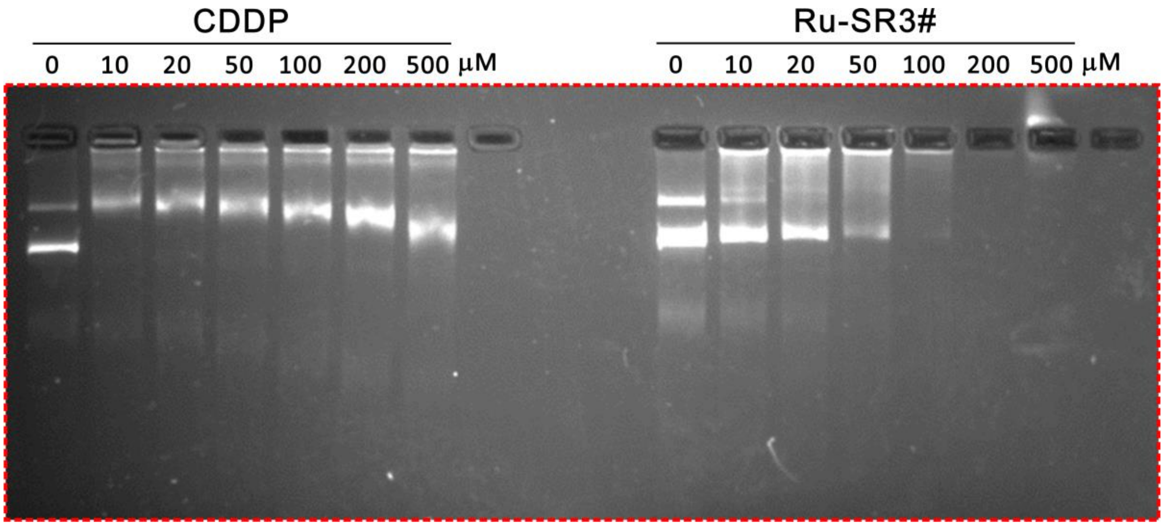

Figure S14: The combination capability of DNA molecules with CDDP (left) and Ru-SR3# (right). The recombinant plasmid DNA PGL3-LUC-Nrf2 promoter (0.25 μg) was incubated with 0, 10, 20, 50, 100, 200, 500 μmol CDDP or Ru-SR3# for 1 h, then proceeded to 1% agarose gel electrophoresis at 8 V/cm voltage for 30 min. After staining by SYBR safe DNA gel stain reagent, the image was recorded by FluroChem M imaging system.

5. Body weight of mice

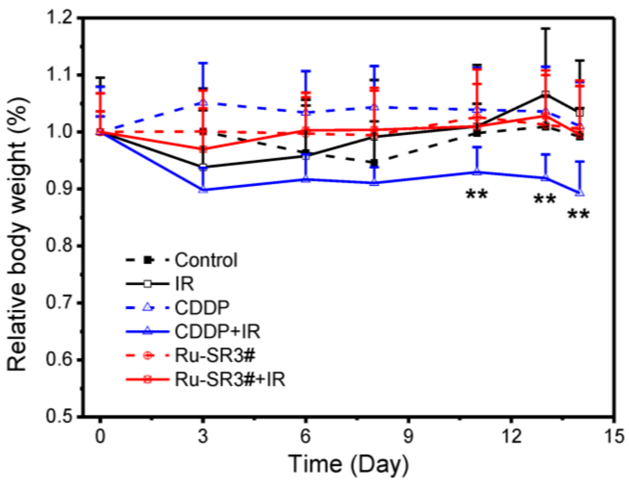

Figure S15: Ru-RS3# facilitates IR to suppress xenografts proliferation in nude mice. 5×10^6 PANC 1 cells were injected into the right rear flanks of Balb/C nude mice to construct the human pancreatic cancer xenografts mice model. The nude mice were undergone chemotherapy and/or radiotherapy as described in Figure 6 (main manuscript). The body weight was measured at around 3 day intervals throughout the treatment. n = 6 per group,**P<0.01, compared with CDDP alone group.

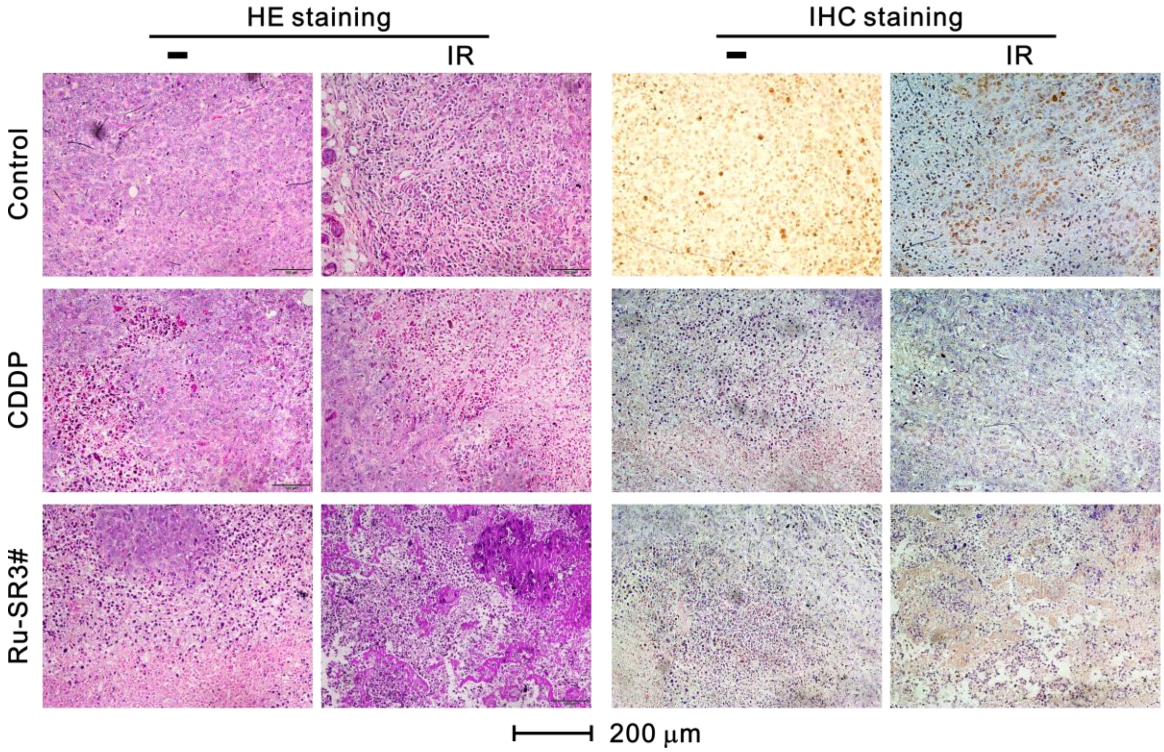

Figure S16: The effect of IR and/or Ru-SR3# treatment on human pancreatic cancer xenografts. At the endpoint of the *in vivo* study, mice were euthanized. Histopathological analysis of xenografts was performed on formaldehyde fixed-paraffin embedded sections via hematoxylin-eosin (H&E) staining (left panels). Also, the immunohistochemistry (IHC) staining of ki67 expression was performed using ki67 antibody (ab15580, Abcam, MA, USA, 1:200) following the user's instruction (right panels). n = 6 per group.
